# I-CARE-An Interaction System for the Individual Activation of People with Dementia

**DOI:** 10.3390/geriatrics6020051

**Published:** 2021-05-13

**Authors:** Tanja Schultz, Felix Putze, Lars Steinert, Ralf Mikut, Anamaria Depner, Andreas Kruse, Ingo Franz, Philipp Gaerte, Todor Dimitrov, Tobias Gehrig, Jana Lohse, Clarissa Simon

**Affiliations:** 1 Cognitive Systems Lab, University of Bremen, 28215 Bremen, Germany; steinela@uni-bremen.de; 2 Karlsruhe Institute of Technology, Institute for Automation and Applied Informatics, 76344 Eggenstein-Leopoldshafen, Germany; ralf.mikut@kit.edu; 3 Institute of Gerontology, University of Heidelberg, 69115 Heidelberg, Germany; anamaria.depner@gero.uni-heidelberg.de (A.D.); andreas.kruse@gero.uni-heidelberg.de (A.K.); 4 Diakonische Hausgemeinschaften Heidelberg e.V., 69126 Heidelberg, Germany; ingo.franz@hausgemeinschaften.de; 5 Media4Care GmbH, 12047 Berlin, Germany; pg@mediadementia.de; 6 Anasoft Technology AG, 44789 Bochum, Germany; dimitrov@technology.de; 7 Videmo Intelligent Video Analysis GmbH & Co. KG, 76131 Karlsruhe, Germany; gehrig@videmo.de; 8 AWO Karlsruhe Gemeinnützige GmbH, 76131 Karlsruhe, Germany; J.Lohse@awo-karlsruhe.de (J.L.); C.Simon@awo-karlsruhe.de (C.S.)

**Keywords:** activation for people with dementia, professional and informal caregivers, technical assistance, intelligent services, recommendation system

## Abstract

I-CARE is a hand-held activation system that allows professional and informal caregivers to cognitively and socially activate people with dementia in joint activation sessions without special training or expertise. I-CARE consists of an easy-to-use tablet application that presents activation content and a server-based backend system that securely manages the contents and events of activation sessions. It tracks various sources of explicit and implicit feedback from user interactions and different sensors to estimate which content is successful in activating individual users. Over the course of use, I-CARE’s recommendation system learns about the individual needs and resources of its users and automatically personalizes the activation content. In addition, information about past sessions can be retrieved such that activations seamlessly build on previous sessions while eligible stakeholders are informed about the current state of care and daily form of their protegees. In addition, caregivers can connect with supervisors and professionals through the I-CARE remote calling feature, to get activation sessions tracked in real time via audio and video support. In this way, I-CARE provides technical support for a decentralized and spontaneous formation of ad hoc activation groups and fosters tight engagement of the social network and caring community. By these means, I-CARE promotes new care infrastructures in the community and the neighborhood as well as relieves professional and informal caregivers.

## 1. Introduction

According to the current state of knowledge, dementia is an incurable progressive disease that ranks first among the age-related fears of people aged 60+ years [1], with close to 50% of all people aged 50+ being worried to develop the disease in the future [2]. The World Alzheimer Report [3] in 2016 estimated that currently around 50 million people are affected by dementia worldwide and that this number will double every 20 years [4] (the World Alzheimer Report is an annual publication with changing foci; its 2020 edition still refers to the cited report as the “most up-to-date global estimate”). A huge increase in care and nursing needs is thus expected. In 2018, worldwide costs will exceed the $1 trillion USD mark [5], 90% of these costs are incurred in the high-income countries. Due to the demographic change, the number of relatives who could take care of people at home will decrease dramatically while the shortage of staff in care institutions will increase, especially in elderly care. In addition to the economic consequences, this will lead to situations where people with dementia (PwD) will not receive a suitable therapy and adequate individual care to the desired extent. The possibilities of interventions and therapies through the adjustments of long-term care insurance, new care structures and the use of intelligent technologies are therefore discussed with explosive force. The latter open up new possibilities to support a self-dependent life in the own home or a self-determined life in care. In particular, technical systems which provide individualized social, cognitive, and motor activation have the potential to delay the negative consequences of the disease and to improve the overall quality of life, e.g., regarding emotional stability, social integration, and self-actualization.

Intelligent information technologies offer an enormous potential for patient-centered and needs-based support of elderly people in general and PwD in particular [6]. Intelligent systems, which adapt to the needs of the patients, coupled with the 24/7 availability of technical systems could offer personalized therapy, which is independent of the amount, time, and expertise of the responsible caregivers. Technical systems may lift off the burden from time-pressured professional and informal caregivers and may foster the integration and engagement of informal caregivers. An important factor to alleviating pressure is the commitment of informal caregivers such as relatives, friends or neighbors, and the caring community. Since dementia is characterized by the progressive loss of mental, social, and physical abilities, the activation and promotion of individual mental and physical abilities as well as social integration are of crucial importance. In this process, informal caregivers play an increasingly important role to ensuring the best possible quality of life for PwD. However, informal caregivers usually have little or no training in looking after people with symptoms of a serious condition like dementia. Technology can support them to improving their skills, reducing stress, and increasing motivation. Such technologies could also support professional caregivers who work on a rotation and do not necessarily know patients well enough to provide individualized activations.

In the collaborative project I-CARE, which was funded by the German Federal Ministry of Education and Research (I-CARE https://www.projekt-i-care.de/ (accessed on 24 April 2021), 2015–2018), seven project partners from science, industry and social service sectors were jointly developing a system that supports the individual needs and potential of PwD. The I-CARE system offers PwD, their relatives and professional and informal caregivers a large variety of activation contents, such as pictures, videos, games, music videos to sing along, biographical photos and quizzes on a conventional tablet PC. I-CARE targets tandems of users, i.e., one person with dementia and one professional or informal caregiver, to jointly use these activation contents. It logs the users’ interactions with the system and evaluates automatically their responses based on facial expressions, spoken interaction, movements, and biosignals. In addition, caregivers can contribute their assessments of each individual activation content. Through this information, the system learns over time the individual needs, preferences and daily conditions of the users with dementia by means of machine learning methods. It subsequently adapts to them by proposing suitable activation contents using a recommendation system. An additional benefit of the learning activation system is that it enables the acquisition of anonymized statistics regarding long-term usage and reception of different activation paradigms. The results will support researchers and practitioners in the field to systematically assess the impact of technology for PwD along various criteria such as the acceptance, efficiency, and usage of activation contents.

**Figure 1:**
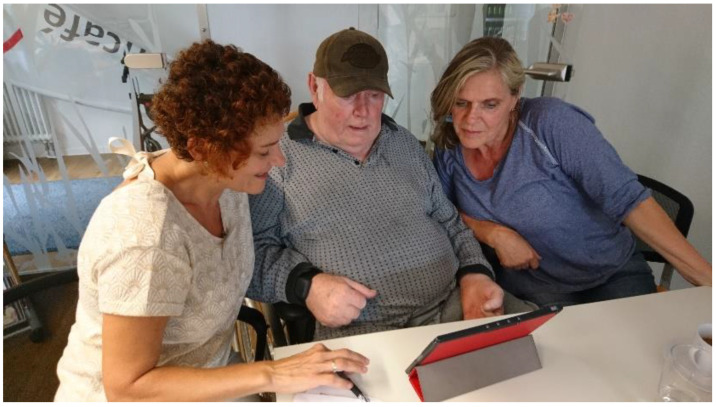
I-CARE tandem session session (Source: © AWO Karlsruhe gGmbH–a written informed consent was obtained from the individuals for the publication of this image).

I-CARE is designed for use at home, in the neighborhood, and in nursing homes where volunteering caregivers such as friends, relatives and neighbors seek ways to effectively and efficiently activate persons to be cared for without the need to be specially trained. Therefore, the I-CARE project aims to provide technical support for a decentralized and spontaneous formation of ad hoc activation groups (Figure 1). This technical support is based on the I-CARE platform. It includes a learning and adapting activation system, individualized activation content, as well as technical components for networking among all participants. With these features, the I-CARE system intends to involve the caring community more tightly in the care of PwD. In particular, the I-CARE system promotes and complements personal care rather than substituting it. The technical backbone of I-CARE allows eligible participants to keep track of the current state of care and of its users and offers activation content that seamlessly connects to previous sessions. In this way, I-CARE can help to set up new infrastructures for care in the community and to relieve caring relatives.

### 1.1. Research Questions

In this paper, we present the I-CARE system in an overview of all its components and how it was administered during a long-term user study. We investigate the following research questions regarding I-CARE:

(1) Which usage patterns can we observe with regards to content categories over time and over different users?

(2) Can the I-CARE system recommend well-matching suitable new activation content from a tandem’s usage history and biographical information?

(3) What type of (cognitive, social, or affective) responses can we observe during tandem sessions with the I-CARE system?

(4) What is the long-term assessment of the tandem partners (PwD and caregivers) of the I-CARE system and its impact?

### 1.2. Ethical Guidelines and Key Objectives

To bring a system for PwD and their caregivers to the field, it is important to consider ethical aspects and to make these understandable and operationalizable for all people in the interdisciplinary consortium, including engineers and computer scientists. Our process (which in many aspects can be generalized to other use cases) to achieve this goal was as follows: To discuss, define and evaluate the ethical guidelines, guiding principles and premises for dealing with the vulnerable group of PwD, a number of workshops were conducted and documented by I-CARE partners [7]. These workshops brought together various professional perspectives from multiple disciplines, including scientists in the field of technology assessment, experts from senior citizens, care and counseling centers, representatives and volunteers from dementia initiatives and networks, plus all I-CARE project partners. In order to promote a change of perspective with a focus on PwD, a dementia obstacle course and the visit of nursing homes was organized for all participants. Over the course of these workshops, particular attention was paid to the data protection concept as well as on the education and support of PwD and their caregivers. Furthermore, several I-CARE partners jointly developed information as well as training materials based on literature research and the evaluation of expert interviews conducted with a focus on technical support of activation during the project; they also wrote the overall concept of project participation for consideration by the Ethics Committee. Partners from social services dealt with the creation of a demand-oriented concept for the neighborhood access, training of project participants considering their resources and deficits [8]. Furthermore, multimodal training materials were developed and iteratively improved according to the feedback of the technical project partners.

The resulting ethical guidelines and guiding principles created in I-CARE and approved by the Ethics Committee of University Heidelberg was published on the project website and made widely available as hand-rails and resources for future research and development of technical systems for PwD.

A major guiding principle was to actively involve participants in the design and selection of the I-CARE activation content. For this purpose, technically operationalizable guidelines were defined in cooperation of gerontology scientists, field experts and industrial partners. As part of the guidelines, a theory- and practice-based concept was developed to complement and diversify the existing activation content, which addresses long-term memory, touches islands of self [9], highlights emotional support and relatedness through the caring community, and focus on socio-spatial worlds. Examples for such guidelines are: using slow and clear language (in sound and video), avoiding stereotypes of old people and infantilization, and including concrete anchors within the content that are recognizable and stimulate discussion between the tandem partners.

## 2. Related Work: I-CARE in Comparison to Other System for People with Dementia

The technical support of PwD is becoming increasingly important due to a variety of factors: the demographic change with a growing number of people in need of care, the rising number of single and childless households along with more mobility and longer distances between parents and adult children, and the shortage of skilled caretakers. Innovations in technology are applied to various areas, i.e., screening for early indicators of dementia as well as in the planning, implementation, and evaluation of intervention measures.

### 2.1. Technical Systems for People with Dementia

While there are currently no preventive measures known that avert the onset of neurodegenerative dementia, nor therapeutic intervention that can prevent or cure the progression of the disease, studies show that the symptoms can be influenced by pharmacological and psychosocial interventions [10,11,12,13] and that the progression of the disease can be delayed. Decisive for the ability to modulate the intervention is to recognize the symptoms as early as possible. Consequently, technical screening systems have been developed that monitor relevant indicators of dementia in the context of casual testing, and to analyze and document trends over time in order to providing the interpretation to clinicians for final diagnostics. Technical systems could thus support a regular low-threshold and nationwide screening for early signs of dementia, which currently cannot be offered due to cost, time, and lack of human resources. One example for low-threshold markers is the spoken language ability that has been shown to be a strong indicator of cognitive abilities [14,15]. As the most important means of communication, speech is used daily to exchange information and to form relationships. Recent findings imply that automatically extracted acoustic and linguistic features of spoken speech indicate the transition into dementia years before the clinical diagnosis [16].

Besides support for diagnosis, wearable and hand-held assistance systems support PwD in structuring tasks [17], in communication [18], navigation [19] and social integration [20] or in training motor skills [21] and encourage physical activity [22]. The category of autonomous assistance systems includes intelligent mobility trainers [23], interactive puppets [18,24], service robots [25], and humanoid robots on wheels such as Pepper (Aldebaran/Softbank) or on two-legs such as Asimo (Honda) and T-HR3 (Toyota). A systematic overview of technical systems for people with cognitive impairments is given by [26], in which they review 91 studies showing that such systems have been used to effectively support cognitive functions related to attention, calculation, emotion, memory, experience of self, as well as higher level cognitive functions like planning and time management. One important category of technical systems for PwD are systems for physical, cognitive, and social activation to support the aforementioned psychosocial interventions and to improve quality of life.

### 2.2. Technical Systems for Activation of People with Dementia

Activation systems are targeted to the individual stimulation of remaining physical, cognitive, and social resources of a user with dementia. Examples of such activation consist of affective and cognitive stimulation with biographical episodic memories of a person, which strengthen the social interaction and self-realization that can cause positive emotions [27]. Furthermore, serious games were designed for physical activation [22] and social activation is targeted by puppets like Elisa [24], cuddly robots like Paro [28], and derivatives like JustoCat (Robyn Robotics AB, Sweden) and by hand-held devices like Media4Care [29], the Asina-Tablet (Borowski IT, Germany) and Doro Experience (Doro, Germany), to name a few.

Many current systems for activation of PwD build on standard tablets and use the available apps to serve videos, images, or other media, as tablets provide high usability, are portable, and do not require a lot of setup or maintenance. For example, Tyack et al. present art on a tablet as a “well-being intervention” [30] in interaction between informal caregivers and PwD. They could show positive tendencies on several well-being metrics. Beyan et al. [31] used natural user interfaces to provide technically supported reminiscence therapy with high usability, leveraging information from available rating databases to select content. Ryan et al. [32] used multimodal content on a tablet for reminiscence therapy together with informal caregivers, such as family members. They also personalized the presented content manually and found that the intervention could help to strengthen relationships and to discover remaining strengths and abilities of the PwD. As an alternative to manual curation of personalized content, Naini et al. [33] investigated the automatic collection through identification of memorable social media posts with machine learning. Foong et al. [34] stressed the importance of considering the role of the volunteering informal caregivers and supporting them through tablet-based guides and material. Gilson et al. [35] showed in a study across more than 1000 sessions that interventions with tablet-based activation through music and videos had a positive effect on the users’ mood after the session. Westphal et al. [36] developed a non-competitive game tailored towards people with dementia reminiscent of the Tangram puzzle.

Other systems explore platforms beyond tablets to perform activation for people with dementia. Nikitina et al. [37] use speech-based, conversational agents to engage PwD in interaction for stimulation. Their system not only presented activation content, but also attempted to moderate its presentation. Sas et al. [38] use wall-sized display as alternative way for content presentation (with a focus on sensory stimulation) on a large scale and could show a high engagement. Some activation systems use music or tangible devices to create activation paradigms which stimulate many senses. These systems require specific hardware to provide a specific form of activation but are able to provide a unique experience. For example, Huber et al. [39] created several prototypes of tangibles for reminiscence therapy, such as a pyramid or a drawer with multimedia output. The devices were able to trigger meaningful physical, cognitive, and social interactions with the device and others. Thoolen et al. [40] created a system for using music cues in the house to trigger meaningful personal memories, including a mixture of wearable, tangible, and ambient components. Independent of the technical platform, activation systems demand highly sophisticated “intelligent” software components since learning algorithms and methods are required that allow for the acquisition and learning of knowledge about personal preferences, daily form, and available resources to automatically adapt the system to individual personal traits and dynamical changing daily conditions.

### 2.3. Studies of Activation Systems in the Field

Other research has taken technical systems targeted at PwD to the field. For example, Zafeiridi et al. [41] investigated a web-based platform for PwD and their caregivers, with a focus on communication and information. In a 1-week user study, they investigated usability and user satisfaction to identify guidance for further developments. A similar platform is investigated by Hattink et al. [42]. They used a mixture of short observations and interviews to evaluate a platform or peer-to-peer exchange and information provision for PwD and caregivers. They found that the system was perceived as usable and useful in general, and furthermore identified several recurring obstacles which users faced. Torkamani et al. [43] performed a long-term user study on a system (ALADDIN) for education and remote care. They showed significant improvement in the quality of life of the caretakers in contrast to a control group. Astell et al. [44] describe the process of development over 7 years for a multimodal computer system (CIRCA) to facilitate communication between PwD and their informal caregivers. They identify a number of important issues, from the need for user-centered development, the relevance of continuous consent, the careful selection of content, and the need to capture verbal and non-verbal responses during system use; many of these aspects inspired the development process of I-CARE.

### 2.4. Summary of the State-of-the-Art

The aims in research and development of technical systems in general are to improve usability and ease of use, optimized compatibility, and retrofitting, as well as user-centric and age-related appropriateness. Many studies assessed the state-of-the-art (e.g., [45,46]), and indicate that currently used systems still consist of basic technologies ranging from electronics and microsystems technology, software engineering, data, and knowledge processing as well as communication technologies, be it individual components and devices, networking solutions or middleware solutions. Lauriks et al. [47] summarize available Information and Communication Technology solutions with respect to the needs of PwD, i.e., along the dimensions of obtaining generalized and personal information, coping with symptoms of dementia, maintaining social contact and company, and enhancing feelings of safety.

Despite the increasing number of technical systems in research and development, to the best of our knowledge there are only very few activation systems available yet that are tested in the field for extended periods of time (i.e., most systems are evaluated in the laboratory or in a small number of supervised sessions). In comparison to most of the presented studies, we here concentrate on a system which is designed for individualized activation in tandem situations (like CIRCA), compared to information or service provision. We also focus on relatively intensive long-term use (like ALADDIN) with multimodal observation, personalization, and individual support. While this approach raises the bar for participation and limits the number of potential participants, it allows us to induce meaningful technology-moderated human-human interactions in uncontrolled conditions.

## 3. Materials and Methods

I-CARE is a hand-held activation system that allows professional and informal caregivers to perform a cognitive, physical, and social activation of PwD in tandem activation sessions [48]. For this purpose, the I-CARE system includes a tablet application that is very easy to operate via an intuitive user interface to present activation contents. While the current interface uses German as an interface language, the extension to other languages is very straightforward due to the modular system design. Events in the activation sessions (such as content selection or quiz responses) are securely stored and managed on a backend system for authorized users and devices. These data are used by the I-CARE system to learn over time about the individual needs and resources of its users to personalize the activation contents. In addition, storing and retrieving past events allows for ad-hoc activation sessions.

### 3.1. I-CARE Activation Content

**Figure 2:**
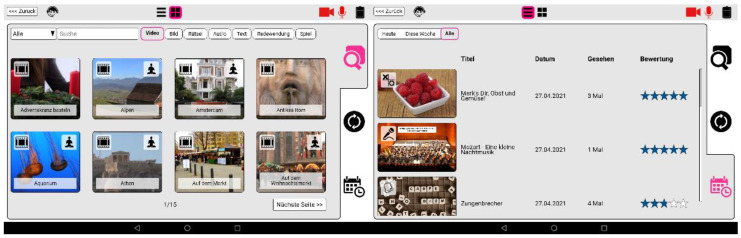
I-CARE Activation Contents (left-hand side) and ratings (right-hand side).

Activation topics (see Figure 2) were chosen and edited to be suitable for conversations among, i.e., they were designed and selected to spark both parties’ interests. This was achieved by a triangulation between personal and biographical themes and materials, events that were formative for the generational belonging, and interesting to people of similar age such as places where people have lived or visited. Furthermore, activation topics were preferred that presented an everyday life perspective to make them more conceivable, to connect more easily to the biographic background, and increase their effectiveness. Topics related to trauma of taboos were considered carefully and chosen rarely. Therefore, the activation concept respects the users as people in their later stages of life and especially considers the risk of a dementia diagnosis. In general, activation contents were selected to minimize obstacles to get the conversation started. Among the most important aspect for activation topic selection and presentation was to provide a non-stereotyped, non-essentialist, diverse picture of elder people and persons with dementia. Therefore, the activation units were designed to represent the highest possible variety and richness regarding fields of interests, social backgrounds, ways of life, moral concepts, as well as cultural and regional socialization, to name a few. Also, special attention was given to different mundane lifestyles of the recent past, i.e., they should be relevant for an elderly audience, possibly be part of their biography and not surrounded by a nostalgic aura. We made sure to make the content accessible and enjoyable for PwD, using the fact that resources and competences of persons with dementia are best stimulated, when processes are shown comprehensibly sparing complex chains of associations.

One industrial partner provided the company’s existing rich selection of activation content, consisting of videos, pictures, karaoke songs (audio), music, games, as well as short stories (textblocks), quizzes, and proverbs [35]. In addition, they produced customized content in terms of about 160 activation films, 100 text and photo quizzes as well as 52 picture series with 10 images each, fitting the aforementioned production criteria. Another industrial partner advanced selected contents such as text quizzes by speech processing technologies to allow for voice-driven quiz question and answering. The implementation of all activation paradigms as web-based applications allows for a flexible adjustment and extension of the user interface. One example of this approach is the generic game interface, which enables the seamless integration of generic interactive content into the logging and content selection mechanisms of the I-CARE system.

The selection and presentation of content was iteratively reviewed and improved in an interdisciplinary working group with both insights from theory and practice. To reach the individualization goal of I-CARE, a cohort-based approach was used as initialization. As cohort parameters, the date and place of birth were applied. This resulted in three thematic blocks for the selection of contents, namely general material of appealing topics, cohort-adapted material such as historical events, celebrities, and known persons from the relevant periods, as well as personal and local material provided by the participants, caregivers and the community. A dedicated web interface was created to allow authorized people the upload of personal and local material, permission provided.

### 3.2. I-CARE System Architecture

**Figure 3:**
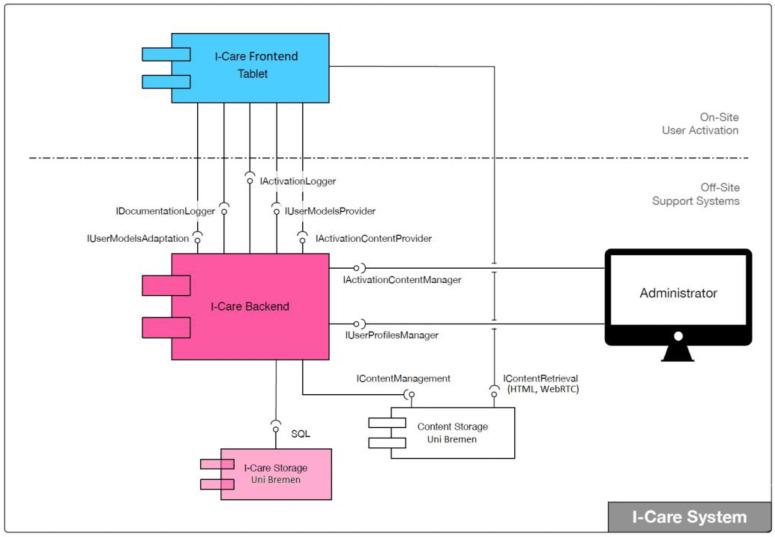
The I-CARE system architecture.

The design and development of the I-CARE system architecture was carried out by an industrial partner, targeting maximal reliability, security, and extendibility. For the implementation of the overall solution, a distributed architecture was designed, which consists of two components (see Figure 3), namely: A frontend application, which runs on a tablet PC, presents the I-CARE content, and annotates the activation sessions, as well as a backend application, which handles centralized data management and administration.

For the frontend application, a service-oriented architecture (SOA) was chosen, which allows for parallel development work by the individual I-CARE partners. It runs on a standard Android tablet PC and establishes an encrypted access-controlled communication with the backend via Wi-Fi or mobile Internet. This design allows to flexibly sharing and exchanging tablets among users. This is an important feature for common use cases in I-CARE, i.e., when one person with dementia is cared for by different caregivers using different tablets or when one caregiver uses the same tablet when working with different PwD throughout the day.

The frontend system on the tablet handles several tasks. First, it is responsible for the graphical user interface which presents the activation content that it retrieves from the backend. Second, it logs the history and events of all activation sessions and third, it transfers the collected session data to the backend system for storage and subsequent data analyses. To perform these tasks, the frontend coordinates and controls intelligent services. By design, the architecture allows for the flexible and scalable integration of services that collect contextual information (e.g., facial expressions, tablet movements, etc.) during the activation sessions and thus provide automated annotations in the form of event logs. This allows the implementation of long-term analyses that are periodically performed offline. The intelligent services themselves are not responsible for the logging nor do they necessarily have to communicate with the backend. For the development of intelligent services, an Android-based software development kit was implemented and provided to all I-CARE partners.

The backend provides the activation content. For this purpose, an administrative web user interface has been implemented, which allows to edit and annotate the content. This includes both general contents provided through the project consortium and individual content provided by users and volunteers. Furthermore, the user account and rights management are carried out on the backend. Authorized people may create and manage biographical information for users. Also, an essential task of the backend is the storage and administration of the event logs associated with each activation session. Depending on the user role, data can be viewed in the backend. Furthermore, the strict privacy and data security policies outlined above, which the entire consortium has committed to comply with have been implemented with the OAuth 2.0 standard for authorization and authentication in I-CARE.

### 3.3. I-CARE Intelligent Services

Intelligent services provide information about the individual activation sessions in the form of user actions and reactions, and context information. For example, it stores how many and which contents were selected by the user, how these contents were rated, and how the user responded to the content. To do so, the I-CARE tablet documents the users’ system interactions in log files containing event lists from each activation session, i.e., the beginning and the end of the activation session, the detected emotions of the user, the explicit evaluation of the content by the user, as well as meta-data in the form of biographical pseudonymized data. Currently, five intelligent services are integrated into the I-CARE system as described in detail in this section.

#### 3.3.1. Annotation and Assessment of Interaction Events

At the beginning of each activation session, the daily condition of the user is requested and logged. After presentation of each single activation content, the user is asked to give a personal review of the content. This explicit assessment is given by a smiley-rating complemented by optional voice comments. Although these explicit assessments are currently performed manually and thus do not require any intelligent algorithm, they are fused with the implicit assessments derived from other modalities like facial expressions, biosignals, and interaction events (see below).

#### 3.3.2. Facial Analysis and Emotion Recognition

One industrial partner developed components for facial analysis based on video data [49,50], and integrated their software into the I-CARE tablet system. During an activation session, facial analysis estimates real-time head posture and facial expressions from volunteering users’ camera data. The video recording function and training options are integrated into the facial analysis module on the I-CARE tablet, and the functionality can be turned on or off via a setting dialog. In order to be able to recognize users by their face, a person model is trained at the beginning of the first session with the I-CARE tablet. During training, features are extracted and collected as long as a face can be detected in the video and landmarks can be identified. After successful training, the model is stored in the associated user profile and reloaded for the next activation session in which this person reappears.

Based on a mimic analysis, three concepts for emotion recognition were established, implemented and evaluated in an exploratory study: the identification of prototypical emotional categories [51], the estimation of continuous emotion dimensions, and the Facial Action Coding System (FACS) [52] based mimic analysis without interpretation of emotions. Based on emotion annotations of 89 I-CARE interview videos, 16 prototypical emotion classes were selected, with the four most frequent classes “interested”, “happy”, “thoughtful” and “inattentive”. For the second approach, emotions were classified along the common two-dimensional space of valence and arousal. The results show that valence can be better estimated, which is assumed to be the more relevant dimension for the I-CARE scenario to assess the quality of an activation content. For the third approach, a system for estimating Action Unit intensities of the FACS was implemented, which uses machine learning methods to process and learn textural and geometrical features of the face, similar to those used for valence and arousal estimation. Since the computation of this facial expression analysis is fast and the description of the visual expressions is most powerful, this approach is currently the preferred choice and was integrated into the I-CARE system. In [53], this approach was used to assess emotional engagement from the facial expression during activation sessions. The employed algorithms are based on machine learning; thus, the face analysis accuracy will benefit as larger amounts of data become available.

#### 3.3.3. Biosignal Analysis based on Interactions, Psychophysiological Indicators and Voice

The content preferences of users are learned by the system from explicit assessments and implicit feedback based on the recorded interactions (touch screen, microphone and camera) as well as physical and psychological indicators from biosignals. In addition to the tablet, users were offered an Empatica E4 wristband [54] that measures movement, electrodermal activity and heart rate based on the integrated sensors. The former refers to the physical activity of the user, while the latter two signals are used to assess physical and psychological parameters (e.g., stress, attention, emotions, enthusiasm). In addition, facial analysis provides information on facial reactions to activation content (e.g., smiles, astonishment), while the analysis of voice recordings allows quantitative statements about to which extent the activation encourages interpersonal communication.

#### 3.3.4. User Modeling and Recommendation System

According to I-CARE’s participatory development strategy, participants’ self-determination should be encouraged in the selection of activation content. At the same time, however, neither PwD nor their informal caregivers should be overwhelmed by time-consuming retrieval procedures when searching for particular contents in the large catalogue. To meet both requirements, a self-learning recommendation system was developed. It aggregates biographical, course-related and contextual information from the activation sessions, and successively adapts the content recommendations to individual user preferences as the system is used over time. Using a content-based filtering algorithm [55], semantic similarities based on ConceptNet [56] are combined with an associative memory approach [57].

For individual activation of users who have never interacted with I-CARE, a new user profile is initialized based on biographical information (e.g., on occupation and hobbies) which is entered during the first sessions. Individualization also allows to exclude specific content, which might be considered inappropriate or potentially traumatic. Once this biographical information is provided, I-CARE recommends cohort-specific activation content. After each activation session, the individual explicit actions (e.g., post-hoc rating of an activation content) and implicit scores of the activation content (e.g., aborting an activation content) are stored in the user profile and used to update the recommendations of contents. As the system usage increases, the user profiles get better adapted to the observed individual preferences.

Technically, the recommendation system is implemented based on the Dice Coefficient, which calculates the similarity of two sets of words. We use the Dice Coefficient in two ways: First, we calculate the similarity of a set of tags for one activation content *T_i_* and the user profile *P_u_*, which is the union of all tags of all content rated positively in previous sessions. Second, we calculate the similarity of *T_i_* and the user biography *B_u_*, which consists of tags related to the interests and the biographic background of the user. To derive a joint coefficient from these two scores, we calculate a weighted sum. The weight *w_p_* depends on the number of items in the user’s history. The calculation can be summarized with the following formula:

(1) $$sim\left(T_{i},P_{u},B_{u}\right)=w_{p}sim_{dice}\left(T_{i},P_{u}\right)+\left(1-w_{p}\right)sim_{dice}\left(T_{i},B_{u}\right)$$

This mechanism can be extended flexibly; for example, we add an additional term with high weight for content which fits to specific events in the year, such as Christmas or the user’s birthday.

For recommendation, we then filter out all content with a similarity score above a threshold and which has not been used in the past 24 h. The remaining items are then sorted by average liking. To ensure a certain degree of content type diversity, we perform fitness proportionate selection, i.e., we chose recommendations from the sorted list of each content type, maintaining the original proportion of types in the selection.

To generate the set of tags for each activation content, we allow maintainers of the content database to enter a description of the content in natural language. These descriptions are then filtered for stop words, stemmed to a base form, and mapped to the concepts of the ConceptNet database. This mapping is used to calculate the pairwise term similarity, by taking the inverse of the shortest distance of the two concepts.

#### 3.3.5. Data-driven Analysis of User Interaction

The I-CARE tablet documents system interactions in log files. These contain event lists of individual activation sessions with timestamp, i.e., (a) the beginning and the end of an activation session and individual activation contents, (b) the detected emotions of the user, (c) the implicit and explicit assessment of the content by the user, and (d) metadata in the form of pseudonymized biographical data. Furthermore, a transformation scheme was implemented that aggregates data both as a single activation session, over the course of sessions, and across all sessions.

For retrospective analyses, the characteristics and time series can be extracted from the aggregated datasets and visualized using special visualizations like heatmaps, such that activation sessions can be interpreted in context and over time. Clustering algorithms are used to identify user groups, and correlation analyses are used to create models that allow conclusions to be drawn from activation content and ratings [58]. The data analysis tools for I-CARE have been integrated into the open source MATLAB toolbox SciXMiner [59] and are available upon request. Based on this groundwork, a toolset for semi-automated analysis of usage statistics and user preferences was created. Some of the resulting statistics are presented in the next section to assess the impact of I-CARE in the field.

### 3.4. I-CARE Evaluation & Study Participants

For evaluation, we performed an extended pilot study with a focus on usage statistics, the recommendation system, and subjective long-term assessment. Across all sections of the evaluation, 29 PwD (18 Female, 11 Male) and their tandem partners participated in the qualitative and quantitative studies. The mean age of the participating PwD was 82.2 years (SD = 8.8). For inclusion in the study, participants had to fulfill the clinical criteria for cognitive disorders involving dementia (Alzheimer dementia, vascular dementia, frontotemporal dementia, Korskoff’s syndrome, Delirium superimposed on dementia, or Not Otherwise Specified), ranging from mild to severe. Of these, 25 completed the study; four participants aborted the study due to health reasons or conflicting goals of caregivers. This results in a very good retention rate of 86%. All study participants were fully informed about the study and gave their written consent to their participation (or, if necessary, the legal guardian of the PwD gave formal consent, with the PwD being involved in the decision making as much as possible). The use of all personal data complies with all relevant national regulations and institutional guidelines. The study has been conducted in accordance with the principles of the Helsinki Declaration and was approved by the Ethics Committee of the University of Heidelberg.

## 4. Results

The I-CARE system was evaluated by an extended pilot study in close cooperation among stakeholders, relatives and professionals in the field. The evaluations were carried out based on qualitative and quantitative studies in participants’ homes and in nursing facilities of two social services partners. For the duration of the studies, one participant or one accompanying person volunteered to carry out joint activation sessions in tandem with the participating person with dementia. In addition to the self-initiated activation sessions, twelve expert-guided sessions were attended by the tandem. These expert-guided sessions consisted of three information and training sessions, eight signal data recordings and observation sessions as well as one reflection session at the end of the study. For inclusion in the study, one of the tandem partners had to fulfill the clinical criteria for a cognitive disorder involving dementia according to the ICD-10, ranging from mild to severe (the degree of severity was often not documented in the diagnose or unreliable in an old diagnose).

During the activation sessions, the following data were collected: (1) personal information for setting up the individualized tablet profile and incorporation of personal materials, (2) wish lists to document the needs and preferences of the relatives or accompanying persons, (3) free-form notes of observations, (4) biosignals data recorded from microphone (voice), camera (face), and touch screen (tablet interaction) along with physical (movement) and psychological indicators (electrodermal activity and heart rate) from an Empatica E4 wristband, (5) manual annotations and observations with respect to emotional reactions, and (6) a final survey questionnaire.

This section may be divided by subheadings. It should provide a concise and precise description of the experimental results, their interpretation, as well as the experimental conclusions that can be drawn.

### 4.1. Usage Statistics

**Figure 4:**
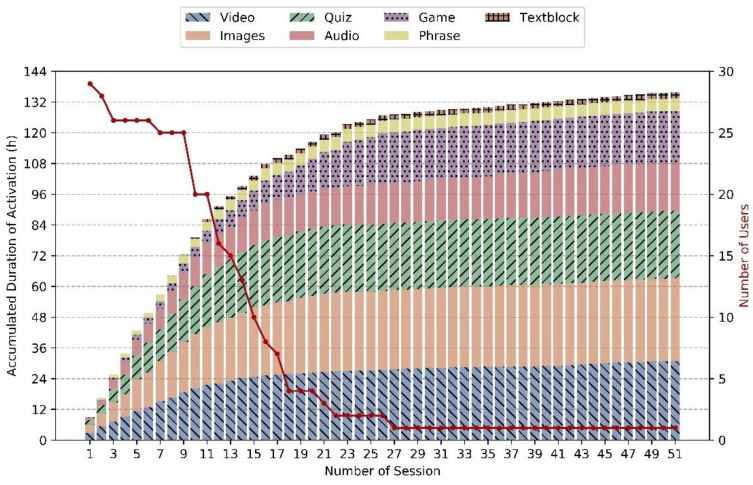
Accumulated duration [hours] of I-CARE activation sessions in the evaluation study.

Almost 400 activation sessions were recorded, with an approximate length of 15 min per session. Half of these sessions were carried out unaccompanied, i.e., self-initiated and without technical support. So far, 25 users have participated in at least 5 sessions, 20 users in at least 10 sessions, and one user has participated in more than 50 sessions. Figure 4 shows the sum of the activation duration over all sessions, broken down into seven content types. It indicates how the content spreads across the categories of videos, quizzes, games, stories (text blocks), images, audio, and phrases like German proverbs or traditional sayings. The distributions vary between sessions, with major proportions of visual material and quizzes. On average, participants started 5.9 activations within a session (standard deviation 6.3, median 5), including a few very long sessions with up to 67 activations (with pauses).

In the pre-study, the tandems were established for a period of 6 months. In practice, this long period led to some tandems leaving before the study was completed. Persons with dementia had to quit mainly for health reasons, and accompanying persons stated that the joint activation sessions plus additional weekly meetings with the evaluation team were too time-consuming. This long-time commitment for 6 months was, in addition to the reservations with respect to recordings of personal data, the biggest hurdle in the acquisition of study participants.

### 4.2. User Preferences

**Figure 5:**
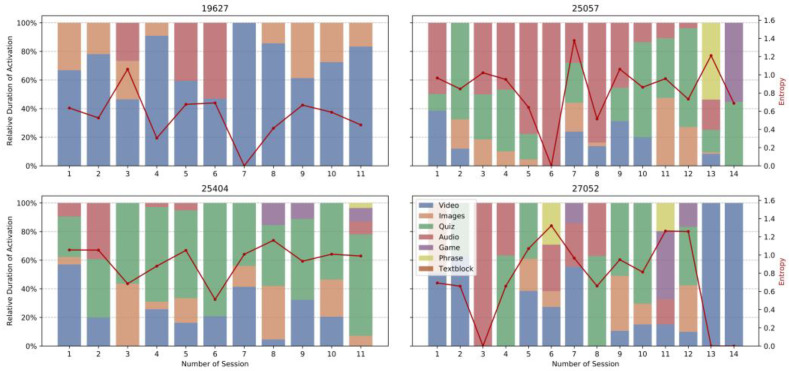
User Preferences over time of four individual tandems. Relative duration of activations as color bars and entropy as red line.

Figure 5 shows the distribution of activation types over the course of all sessions for four individual tandems. Although the number of users does not allow for a statistical significance statement, this side-by-side comparison indicates temporal dynamics and individual differences in the selection of activation types with no clear pattern of general preferences. This observation supports our approach to develop a recommendation system, which learns preferred types while ensuring a minimal heterogeneity and novelty of recommended items. The red lines in the plots denote the diversity of types throughout the sessions (measured as entropy). Entropy is defined as, where the *P_i_* denote the relative frequency of content types:

(2) $$S=-\underset{i}{\sum }P_{i}*\log(P_{i})$$

We observe no convergence to a single preferred item type. For all shown tandems, entropy stays on a similar level throughout the progression of the study, showing that most tandems prefer a variance in the selection of activation types within one session.

**Figure 6:**
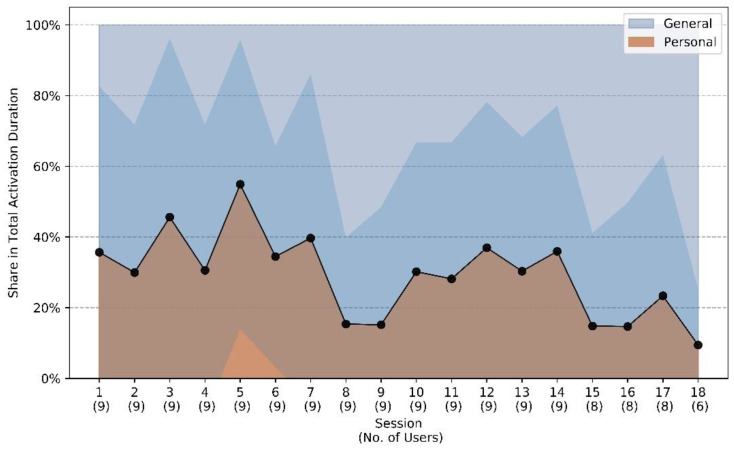
Content personalization with duration of usage with large interpersonal increases variances.

**Figure 7:**
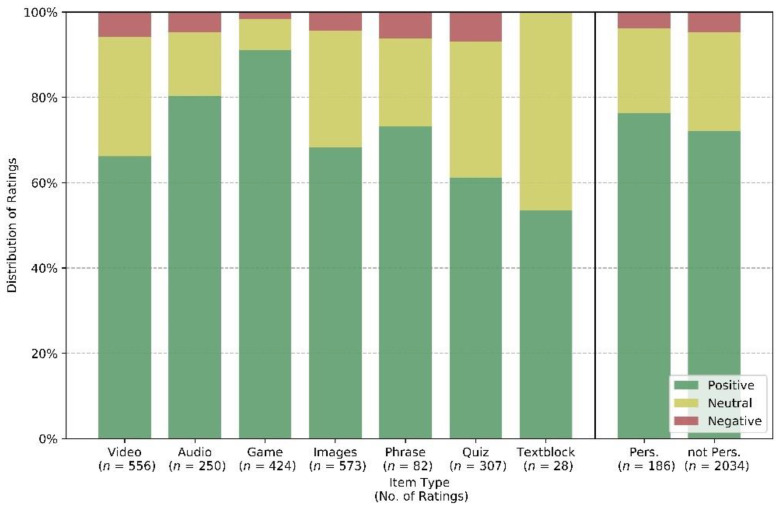
All types of content get mostly positive assessments, with games and audio achieving best marks on average.

Figure 6 shows how often personal content is selected compared to general content as a function of time over the course of the study. This shows that personalized content takes an average share of more than 50% at the peak, but there is a downward trend towards the end of study participation, as personalized content is rarely renewed after the initial provision, while the general content library increases. Figure 7 shows that participants give mostly positive assessments to the presented material, with games and audios getting the best marks on average. The two right-most bars indicate that personalized contents achieve slightly better ratings, with more positive and fewer negative ratings compared to non-personalized items. However, the differences are not statistically significant and more than a third of the personalized content is not rated positively, implying that individual ratings not only assess the content but also take situational response into account.

**Figure 8:**
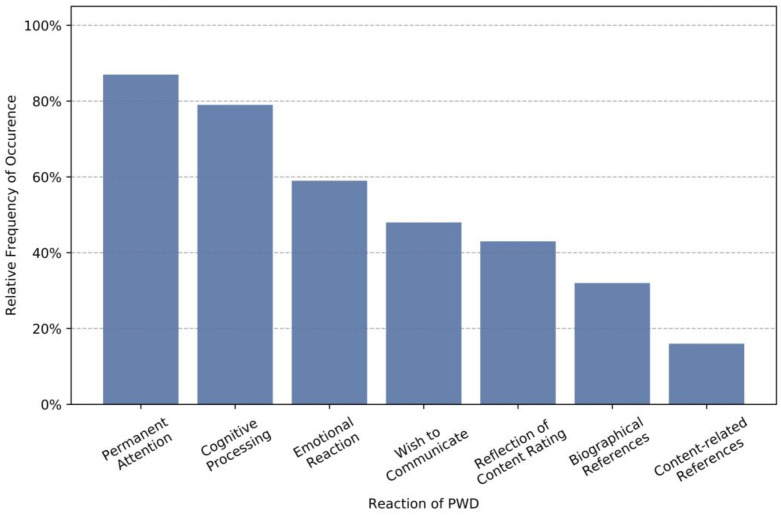
Relative frequency of observed responses to activation material.

Figure 8 shows how participants responded to the presentation of stimulus material, as derived from the observation of a subset of sessions (747 activation trials). The observation focuses on the tandem partners with dementia and shows for more than 80% of the cases that users paid sustained attention to the presented material and close to 80% showed noticeable signs of cognitive processing of the material. This implies that the material is actively examined in most cases and not just passively consumed. In close to 60% of all cases, participants show emotional responses to the material and for nearly half of the cases, participants spontaneously expressed a wish to communicate with their tandem partner. Both observations indicate that the selected topics allow a multifaceted stimulation, on a cognitive, emotional, and social level. One of the most important topics in discussions seemed to be the evaluation of the material, which may have been triggered by the explicit rating requested after each activation. Interestingly, content-related references to the presented material are relatively infrequent. In fact, biographical references were observed in twice as many cases. This underlines the importance of cohort-specific or personalized material to which users can relate to.

### 4.3. Recommendation System Evaluation

**Figure 9:**
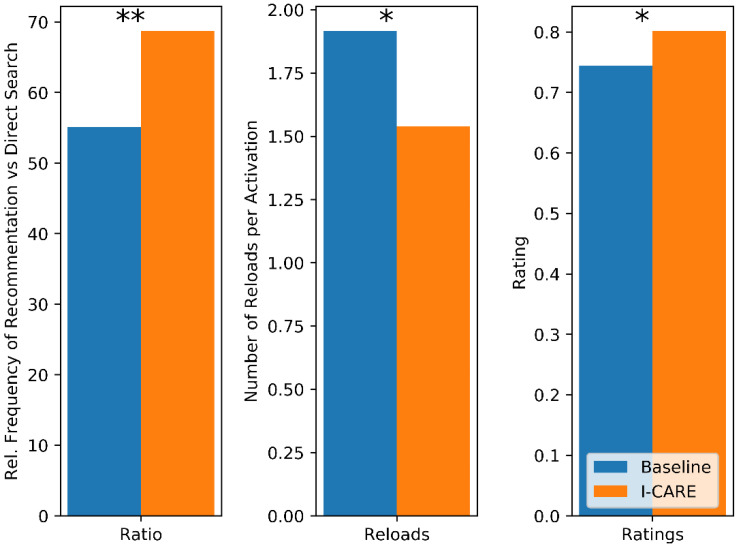
Different performance metrics for the comparison of Baseline Recommender and I-CARE recommender. (*) denotes a significant difference (*p* < 0.05), (**) denotes a highly significant difference (*p* < 0.001) difference.

To study the effect of the I-CARE recommendation system, we included an A-B test for a subset of sessions between the I-CARE recommender system and a baseline system that suggests activation content randomly based on the generally most popular content. At the beginning of each session, the system decides between both types of activation. Figure 9 shows how both recommending systems compare against each other according to three different metrics. First, we look at how often users select activation content from the recommendations compared to a direct search. We expect a higher value for a recommender which is considered more useful. While 55% of all content is selected from the recommendations in the Baseline conditions, it is 69% in the I-CARE condition. The second metric measures the number of reloads triggered on average per activation. Here, a lower value is preferable. The observed values are 1.9 for the Baseline condition and 1.5 for the I-CARE condition, i.e., participants are more likely to accept the early suggestions by the I-CARE recommender. Finally, we look at the average rating given to the contents recommended by the two different systems, ranging from 0 (worst) to 1 (best). Here, we observe an average score of 0.74 for the Baseline and 0.8 for the I-CARE condition. For all three metrics, the difference between Baseline and I-CARE system are significant (*p* = 0.0006, 0.02 and 0.02) using a one-sided, paired t-test across participants.

### 4.4. Questionnaire

**Figure 10:**
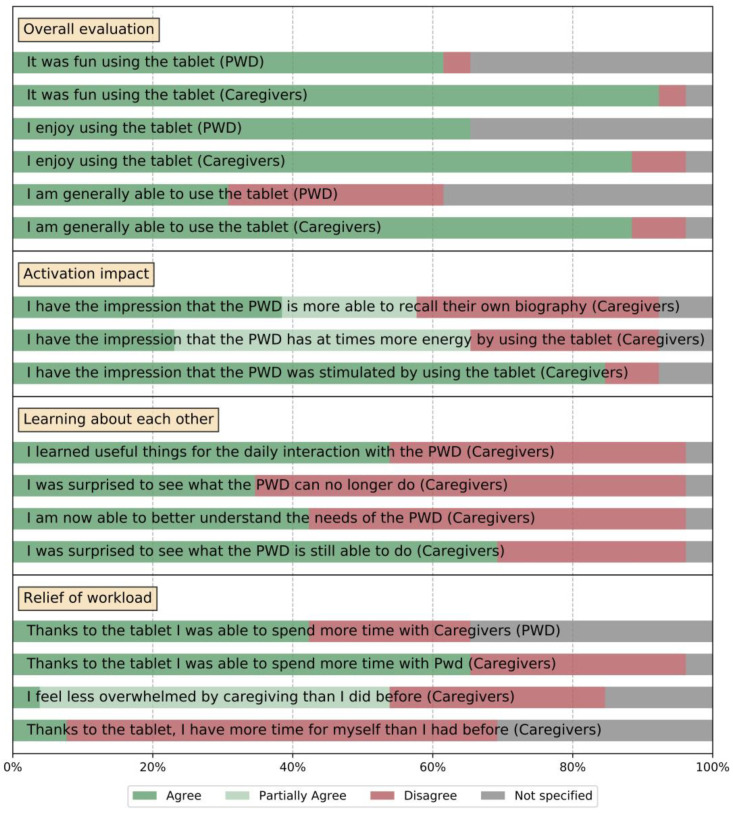
Responses to final questionnaire at the end of the evaluation (*n* = 25) for persons with dementia (PwD) and Caregivers.

After the conclusion of the study period, each tandem filled out a concluding questionnaire, which covered the experience of the full study duration of three (or six) months of system use. Besides a qualitative component with opportunities to give suggestions for additional activation content, a central part of the questionnaire was a quantitative component that covered the impact of the system on the tandem’s quality of life, with regard to several different aspects. These were covered by a sequence of Likert scales with statements to which participants could agree, partially agree (only available for some statements which relate to the occurrence of specific events over the course of participation), or disagree. We report combined results from all parts of the study, which covers a broad range of I-CARE use cases at home and in nursing centers, as well as with informal and professional caregivers, shown in Figure 10. The first block of statements related to an overall evaluation of the system. The large majority of all caregiving participants reported that the use of the I-CARE system was “fun” (>90%) and that they “enjoyed” (>80%). This number is lower for the participants with dementia; however, this may be because in many cases, these participants declined to answer or were not able to.

Most caregivers reported to feel proficient in the independent operation of I-CARE, which supports our approach to create a complex activation system which can be used by informal caregivers (and often technical novices). When evaluating the more specific effects of the system such as the “activation impact”, more than 80% of all participants reported an activating effect through I-CARE. To a lesser extent, participants also observed a positive effect on biographic memory and general energy. I-CARE involves the joint exploration of personalized content and encourages biographical references to cohort-specific material. Additionally, the joint use of the I-CARE tablet creates interaction situations between both tandem partners, which may be different from other everyday interactions. For both reasons, the use of I-CARE gives both tandem partners the opportunity to learn something about themselves and each other. This is reflected in the fact that more than 70% of all caregivers in the study report to have learnt about the skill reserves of their tandem partners with dementia. For example, one informal caregiver worked twice a week with her tandem partner, a person with dementia, and reported that they were once sitting together in a doctor’s waiting room looking at children’s book when the person with dementia pressed her finger on the objects as if she would expect a window to pop up. Prior to working with I-CARE the lady with dementia had never used a smartphone or PC. Finally, since PwD do not use the I-CARE tablet all by themselves but rather in tandem settings, I-CARE provides an opportunity for both tandem partners to get together more often, which by itself is a gain in quality of life.

## 5. Discussion

This article describes the I-CARE system, which uses intelligent information technologies and intensive participatory design to enable individualized activations of PwD, which do not depend on the number, time budget and level of knowledge of informal and professional caregivers. The I-CARE system was evaluated in close collaboration with participants and professionals based on qualitative and quantitative studies in several facilities in an extended pilot study. Data analyses of user statistics from a longitudinal user study confirm the benefits of an automatic individualization of activation content. Main findings in the study were: (1) A transparent and continuous support of PwD and informal caregivers during the study leads to a high retention rate of participants, even in a long-term study over several months. (2) Usage of the system and the provided content is diverse, between different participants as well as between different sessions of the same participant. (3) the activation through I-CARE proved to be successful, resulting in a broad spectrum of responses, ranging from sustained attention, over cognitive processing, to emotional reactions. Especially cohort-specific or personalized material stimulated biography-related discussion. (4) The I-CARE recommendation system significantly outperforms the baseline algorithm, showing that even from very few data points in a specialized domain, we can successfully leverage information which was gathered from usage preferences and biography. (5) In post-study questionnaires, the overwhelming majority of participants reported the interaction with I-CARE as fun and joyful. Reported effects were activation, learning about each other, as well as meaningful interactions in addition to routine everyday encounters.

There are several limitations of the I-CARE system and the conducted study which should be addressed in future work: The system uses a large variety of sensors and a sizable corpus of activation content to recommend appropriate content for an individual user; however, recommendations are based initially only on the relatively general biographic information and then on one data point per performed activation. This means that the cold start phase of the system, in which the system has little information for a new user, is relatively long and users could opt out before recommendations become tailored towards them. In the future, it would be useful to leverage a larger information base, for example by identifying clusters of user types with specific content preferences. The system could then use small amounts of biographical information and initial information to identify the fitting clusters and use the associated recommendations until more individual information becomes available. The pilot study is limited by the small sample size with a high variance between participants, a result of the longitudinal approach of month-long system use in real-world settings. We consider this a strength of our approach, but it also limits the statistical power of the analysis and should be complemented with additional data from more comparable settings (e.g., only users in living in their own home) and a control group. Such a study could concentrate on a more rigorous statistical evaluation of some of the findings in the present study.

## Data Availability

For privacy reasons, the data in this study is not publicly available.

## References

[1] (2017). Forsa-Umfrage ‘Angst vor Krankheiten’ im Auftrag der DAK. https://www.dak.de/dak/download/forsa-umfrage-1949432.pdf.

[2] Maust D.T., Solway E., Langa K.M., Kullgren J.T., Kirch M., Singer D.C., Malani P. (2020). Perception of dementia risk and preventive actions among US adults aged 50 to 64 years. JAMA Neurol..

[3] (2016). World Alzheimer Report. https://www.alz.co.uk/research/world-report-2016.

[4] Ferri C.P., Prince M., Brayne C., Brodaty H., Fratiglioni L., Ganguli M., Hall K., Hasegawa K., Hendrie H., Huang Y. (2005). Global prevalence of dementia: A Delphi consensus study. Lancet.

[5] Wimo A., Guerchet M., Ali G.-C., Wu Y.-T., Prina A.M., Winblad B., Jönsson L., Liu Z., Prince M. (2017). The worldwide costs of dementia 2015 and comparisons with 2010. Alzheimers’ Dement..

[6] Schultz T., Putze F., Kruse A. (2014). Technische Unterstützung für Menschen mit Demenz: Symposium 30.09.-01.10.2013.

[7] Depner A. Wie kann man das machen? Methodische und ethische Dimensionen partizipativer Ansätze zur Einbindung verschiedener Akteure in die Entwicklung technischer Aktivierungssysteme für Menschen mit Demenz. Proceedings of the AAL Congress.

[8] Lohse J., Simon C. (2017). Individuelle Aktivierung von Menschen mit Demenz—Tablet soll Freude und Neugier an neuer Technologie wecken. Alzheimeraktuell.

[9] Kruse A. (2017). Lebensphase hohes Alter: Verletzlichkeit und Reife.

[10] Vernooij-Dassen M., Vasse E., Zuidema S., Cohen-Mansfield J., Moyle W. (2010). Psychosocial interventions for dementia patients in long-term care. Int. Psychogeriatr..

[11] Van Mierlo L., van der Roest H., Meiland F., Dröes R. (2010). Personalized dementia care: Proven effectiveness of psychosocial interventions in subgroups. Ageing Res. Rev..

[12] O’Connor D.W., Ames D., Gardner B., King M. (2009). Psychosocial treatments of behavior symptoms in dementia: A systematic review of reports meeting quality standards. Int. Psychogeriatr..

[13] García-Casal J.A., Loizeau A., Csipke E., Franco-Martín M., Perea-Bartolomé M.V., Orrell M. (2017). Computer-based cognitive interventions for people living with dementia: A systematic literature review and meta-analysis. Aging Ment. Health.

[14] Bickel C., Pantel J., Eysenbach K., Schröder J. (2000). Syntactic comprehension deficits in Alzheimer’s disease. Brain Lang..

[15] Bucks R.S., Singh S., Cuerden J.M., Wilcock G.K. (2000). Analysis of spontaneous, conversational speech in dementia of Alzheimer type: Evaluation of an objective technique for analysing lexical performance. Aphasiology.

[16] Weiner J., Schultz T. Automatic screening for transition into dementia using speech. Proceedings of the Speech Communication; 13th ITG-Symposium.

[17] Seelye A.M., Schmitter-Edgecombe M., Cook D.J., Crandall A. (2013). Naturalistic assessment of everyday activities and prompting technologies in mild cognitive impairment. J. Int. Neuropsychol. Soc..

[18] Kuhlmann A., Reuter V., Schramek R., Dimitrov T., Görnig M., Matip E.v., Matthies O., Naroska E. (2018). OurPuppet–Pflegeunterstützung mit einer interaktiven Puppe für pflegende Angehörige. Zeitschrift für Gerontologie und Geriatrie.

[19] Chang Y.-J., Tsai S.-K., Wang T.-Y. A context aware handheld wayfinding system for individuals with cognitive impairments. Proceedings of the 10th international ACM SIGACCESS conference on Computers and accessibility.

[20] Rehrl T., Troncy R., Bley A., Ihsen S., Scheibl K., Schneider W., Glende S., Goetze S., Kessler J., Hintermueller C. The ambient adaptable living assistant is meeting its users. Proceedings of the AAL Forum 2012-Eindhoven.

[21] Fasola J., Mataric M.J. (2012). Using socially assistive human–robot interaction to motivate physical exercise for older adults. Proc. IEEE.

[22] Lin J.J., Mamykina L., Lindtner S., Delajoux G., Strub H.B. Fish’n’Steps: Encouraging Physical Activity with an Interactive Computer Game. Proceedings of the International Conference on Ubiquitous Computing.

[23] Guhl T., Heuer S., Rosales B., Walther S., Schneider J., Schultz T., Putze F., Kruse A., Hg. (2014). Entwicklung eines Mobilitätsassistenten für Eingeschränkte Personen–Hintergrund, Status und Möglichkeiten der Kooperation.

[24] Dimitrov T., Kramps O., Ressel C., Könen S., Matthies O., Habibi A., Matip E.M., Polzehl T., Voigt-Antons J.N., Heutelbeck D. (2018). “OurPuppet”–Entwicklung einer Mensch-Technik-Interaktion für die Unterstützung informell Pflegender. Zukunft der Pflege Tagungsband der 1. Clusterkonferenz.

[25] Graf B., Reiser U., Hägele M., Mauz K., Klein P. Robotic home assistant Care-O-bot^®^ 3—product vision and innovation platform. Proceedings of the 2009 IEEE Workshop on Advanced Robotics and its Social Impacts.

[26] Gillespie A., Best C., O’Neill B. (2012). Cognitive function and assistive technology for cognition: A systematic review. J. Int. Neuropsychol. Soc..

[27] Crete-Nishihata M., Baecker R.M., Massimi M., Ptak D., Campigotto R., Kaufman L.D., Brickman A.M., Turner G.R., Steinerman J.R., Black S.E. (2012). Reconstructing the past: Personal memory technologies are not just personal and not just for memory. Hum. Comput. Interact..

[28] Wada K., Shibata T., Musha T., Kimura S. (2008). Robot therapy for elders affected by dementia. IEEE Eng. Med. Biol. Mag..

[29] (2016). Media4Care: Das Tablet für Senioren, Menschen mit Demenz und ihre Betreuer. https://www.media4care.de.

[30] Tyack C., Camic P.M., Heron M.J., Hulbert S. (2017). Viewing art on a tablet computer: A well-being intervention for people with dementia and their caregivers. J. Appl. Gerontol..

[31] Bejan A., Gündogdu R., Butz K., Müller N., Kunze C., König P. (2018). Using multimedia information and communication technology (ICT) to provide added value to reminiscence therapy for people with dementia. Zeitschrift für Gerontologie und Geriatrie.

[32] Ryan A.A., McCauley C.O., Laird E.A., Gibson A., Mulvenna M.D., Bond R., Bunting B., Curran K., Ferry F. (2020). There is still so much inside’: The impact of personalised reminiscence, facilitated by a tablet device, on people living with mild to moderate dementia and their family carers. Dementia.

[33] Naini K.D., Kawase R., Kanhabua N., Niederée C., Altingovde I.S. (2019). Those were the days: Learning to rank social media posts for reminiscence. Inf. Retr. J..

[34] Foong P.S., Zhao S., Carlson K., Liu Z. Vita: Towards supporting volunteer interactions with long-term care residents with dementia. Proceedings of the 2017 CHI Conference on Human Factors in Computing Systems 2017.

[35] Gilson A., Dodds D., Kaur A., Potteiger M., Ford J. (2019). Using computer tablets to improve moods for older adults with dementia and interactions with their caregivers: Pilot intervention study. JMIR Form. Res..

[36] Westphal B.J., Lee H., Cheung N.M., Teo C.G., Leong W.K. Experience of Designing and Deploying a Tablet Game for People with Dementia. Proceedings of the 29th Australian Conference on Computer-Human Interaction.

[37] Nikitina S., Callaioli S., Baez M. Smart conversational agents for reminiscence. Proceedings of the 2018 IEEE/ACM 1st International Workshop on Software Engineering for Cognitive Services (SE4COG).

[38] Sas C., Davies N., Clinch S., Shaw P., Mikusz M., Steeds M., Nohrer L. Supporting stimulation needs in dementia care through wall-sized displays. Proceedings of the 2020 CHI Conference on Human Factors in Computing Systems.

[39] Huber S., Berner R., Uhlig M., Klein P., Hurtienne J. Tangible objects for reminiscing in dementia care. Proceedings of the Thirteenth International Conference on Tangible, Embedded, and Embodied Interaction.

[40] Thoolen M., Brankaert R., Lu Y. AmbientEcho: Exploring Interactive Media Experiences in the Context of Residential Dementia Care. Proceedings of the 2020 ACM Designing Interactive Systems Conference 2020.

[41] Zafeiridi P., Paulson K., Dunn R., Wolverson E., White C., Thorpe J.A. (2018). A web-based platform for people with memory problems and their caregivers (CAREGIVERSPRO-MMD): Mixed-methods evaluation of usability. JMIR Form. Res..

[42] Hattink B., Droes R.-M., Sikkes S., Oostra E., Lemstra A.W. (2016). Evaluation of the Digital Alzheimer Center: Testing usability and usefulness of an online portal for patients with dementia and their carers. JMIR Res. Protoc..

[43] Torkamani M., McDonald L., Aguayo I.S., Kanios C., Katsanou M.-N., Madeley L., Limousin P.D., Lees A.J., Haritou M., Jahanshahi M. (2014). A randomized controlled pilot study to evaluate a technology platform for the assisted living of people with dementia and their carers. J. Alzheimers’ Dis..

[44] Astell A., Alm N., Gowans G., Ellis M., Dye R., Vaughan P. (2009). Involving older people with dementia and their carers in designing computer based support systems: Some methodological considerations. Univers. Access Inf. Soc..

[45] Löfqvist C., Nygren C., Széman Z., Iwarsson S. (2005). Assistive devices among very old people in five European countries. Scand. J. Occup. Ther..

[46] Salminen A.-L., Brandt Å., Samuelsson K., Töytäri O., Malmivaara A. (2009). Mobility devices to promote activity and participation: A systematic review. J. Rehabil. Med..

[47] Lauriks S., Reinersmann A., Van Der Roest H., Meiland F., Davies R., Moelaert F., Mulvenna M., Nugent C., Dröes R. (2007). Review of ICT-based services for identified unmet needs in people with dementia. Ageing Res. Rev..

[48] Schultz T. I-CARE: Individual activation of people with dementia. Proceedings of the 13th biannual conference of the German cognitive science society (KogWis 2016).

[49] Gehrig T., Al-Halah Z., Ekenel H.K., Stiefelhagen R. Action Unit Intensity Estimation Using Hierarchical Partial Least Squares. Proceedings of the 2015 11th IEEE International Conference and Workshops on Automatic Face and Gesture Recognition (FG).

[50] Fischer M., Ekenel H.K., Stiefelhagen R. (2011). Person re-identification in tv series using robust face recognition and user feedback. Multimed. Tools Appl..

[51] Richter M., Gehrig T., Ekenel H.K. Facial Expression Classification on Web Images. Proceedings of the 21st International Conference on Pattern Recognition (ICPR2012).

[52] Ekman P., Friesen W.V. (1978). Manual for the Facial Action Coding System.

[53] Steinert L., Putze F., Küster D., Schultz T. Towards Engagement Recognition of People with Dementia in Care Settings. Proceedings of the 2020 International Conference on Multimodal Interaction.

[54] (2018). Empatica Inc.—ISO 13485 Cert. No. 9124.EPTC. https://www.empatica.com/.

[55] Jannach D., Zanker M., Felfernig A., Friedrich G. (2010). Recommender Systems: An Introduction.

[56] Liu H., Singh P. (2004). ConceptNet—A practical commonsense reasoning tool-kit. Bt Technol. J..

[57] Pröpper R., Putze F., Schultz T. (2011). Jam: Java-Based Associative Memory. Proceedings of the Paralinguistic Information and its Integration in Spoken Dialogue Systems Workshop.

[58] Doneit W., Lohse J., Glesing K., Simon C., Fischer M., Depner A., Kruse A., Franz I., Schultz T., Putze F. (2017). Data-driven analysis of interactions between people with dementia and a tablet device. Curr. Dir. Biomed. Eng..

[59] Mikut R., Bartschat A., Doneit W., Ordiano J.Á., Schott B., Stegmaier J., Waczowicz S., Reischl M. (2017). The MATLAB toolbox SciXMiner: User’s manual and programmer’s guide. arXiv.

